# Computational and materials structural science

**DOI:** 10.1107/S2052252519009114

**Published:** 2019-06-28

**Authors:** C. Richard A. Catlow

**Affiliations:** a Department of Chemistry, University College London, 20 Gordon Street, London WC1 HOAJ, UK; b School of Chemistry, Cardiff University, Park Place, Cardiff CF10 3AT, UK

**Keywords:** computational structural science, materials structural science, editorial

## Abstract

This Editorial discusses recent development in computational and materials structural science as exemplified by recent articles published in **IUCrJ**.

The themes of materials and computation continue to grow and diversify in **IUCrJ**, but with a continuing emphasis on unravelling the structural science of complex functional materials and on developing further understanding of structure–property relations. The recent articles in the journal highlight both developments in technique and approach, as well as the exploration of new classes of system and of problems.

The last year has seen significant growth in structural nanoscience, with developments in the study of alloy nanoparticles of importance in catalytic science The paper of Liang & Yu (2019 ▸), also discussed by Slabon (2019 ▸), uses aberration-corrected scanning transmission electron microscopy (AC-STEM), to provide a structural model of intermetallic AuCu nanoparticles, with a chemically ordered AuCu core encapsulated within a few-atoms-thick Au shell. This type of segregation with core–shell structures is increasingly observed in alloy nanoparticles, as in the earlier work of Gibson *et al.* (2015 ▸). Another fascinating area of nanoscience is illustrated by the article of Chiu *et al.* (2019 ▸), which describes the formation of gyroid-structured Au nanonetworks, fabricated through the development of Au nanoparticles. A schematic of the resulting structures is shown in Fig 1 ▸.

Alloy structural science continues to be a widely explored theme, with computational methods being used by Han *et al.* (2019 ▸) to predict new Heusler alloys and studies of stress-induced detwinning and phase transformations in ternary alloys by Hou *et al.* (2019 ▸). Ternary alloys are also the subject of an intriguing investigation by Martino *et al.* (2018 ▸) of a new compound, Sr_2_Pt_8 − *x*_As, which is prepared by high-pressure synthesis and has a high concentration of vacancies which are incommensurately ordered.

Another growing area is the use of data-mining, machine-learning and high-throughput screening techniques within computational materials science. Nguyen *et al.* (2018 ▸) report a new method to measure the similarity between materials, focusing on specific physical properties, while Jin *et al.* (2019 ▸) describe how a large-scale computational screening exercise can be used to predict new topological materials.

The structural science of disordered materials is a perennial theme. Gao *et al.* (2019 ▸) describe a new theoretical approach to defect generation during deformation – a key factor controlling the mechanical properties of materials; while Rudolph *et al.* (2019 ▸) develop new models for the defect structure of the widely studied γ-alumina, showing that the predominant defects are planar antiphase boundary structures. Subtle modes of disorder are identified by Takada *et al.* (2018 ▸) in the molecular dynamics (MD) simulation studies of the structures of tridymite, where they show, for example, that the structure of HP-tridymite, determined from diffraction experiments, can be described as a time-averaged structure in a similar manner to β-cristobalite, with ‘floppy’ oxygen modes playing an important role.

MD simulations are also used in a study of van de Streek *et al.* (2019 ▸) of phase transitions of a ‘jumping crystal’, with the simulations predicting the structure of the high-temperature structure, which is then verified by refinement against X-ray powder data.

Chemical crystallography, especially of complex ternary or quaternary oxides remains a continuing theme and is well represented by the study of Delacotte *et al.* (2018 ▸) on hexaferrites – an important class of magnetic oxides with applications in data storage and electronics. The structures revealed for these complex ternary oxides include a new hexaferrite stacking sequence, with the longest lattice parameter of any hexaferrite with a fully determined structure. Organic materials also present challenges in structural science, as shown by the study of Jeannin *et al.* (2018 ▸) on one-dimensional organic conductors showing an interesting phase diagram in which the effects of anion ordering and spin-Peierls transition are decoupled; while Huang *et al.* (2019 ▸) present a systematic theoretical study of the conducting and optical properties of a family of aromatic di­imides. This latter work again illustrates the power of computational techniques in probing the properties of complex materials.

In conclusion, the recent articles in the journal show that structural materials science continues to diversify, with computation being firmly integrated in the field. Articles in these areas will continue to be welcomed by **IUCrJ**.

## Figures and Tables

**Figure 1:**
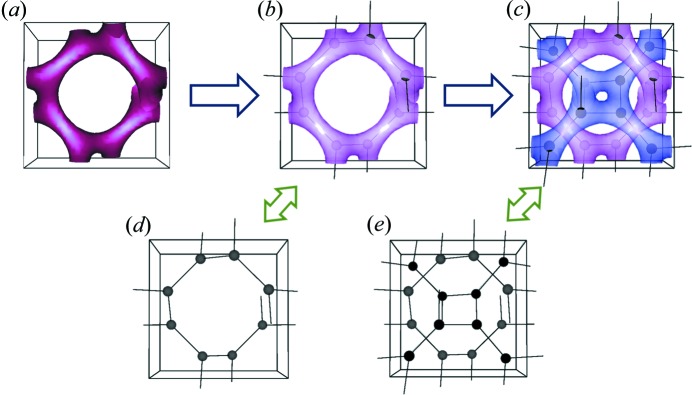
Schematic illustrations of (*a*) the unit cell of a single gyroid, (*b*) the nodes of a single gyroid and (*c*) the nodes of a double gyroid, with the corresponding simplified models (*d*) and (*e*) in the respective unit cells. The effective spheres are featured as nodes for visualization. Lines are used as a guide to the eye [after Chiu *et al.* (2019 ▸)].
